# Dr. John J. R. Patrick

**Published:** 1895-06

**Authors:** A. H. Fuller


### Obituary.

### DR. JOHN J. R. PATRICK.

John J. R. Patrick, D.D.S., died at his home in Belleville, Ill., on the 10th of April, after a lingering illness, at the age of sixty-seven. Dr. Patrick was born in Liverpool, England, February 6, 1828, coming to this country with his parents when fourteen years of age. The family moved to Keokuk, Iowa, where his father, Dr. Hugh Patrick, died in 1847.

After the death of his father, John, with his mother and others of the family, came to St. Louis, where he learned the trade of a goldsmith. He followed this calling for several years, both in St. Louis and New Orleans. He later studied dentistry with his brother, and after attending one course of lectures in the McDowell Medical College, commenced the practice of dentistry in St. Louis.

Dr. Patrick married Miss Jane Johnston in 1853, and removed to Belleville, Ill., where he has resided ever since up to the time of his death. During the war of the Rebellion the doctor served as captain of Company G, One Hundred and Thirtieth Illinois Infantry Volunteers, from January 23, 1863, to January 14, 1865, being mustered out of service upon the consolidation of his regiment with the Seventy-seventh Illinois. At the time of his death, and for several years previous, he was a member of the Grand Army of the Republic and of the Illinois Commandery of the Loyal Legion. For many years the doctor interested himself in the study of palæontology, spending much time and money in this pursuit. He had the Indian mounds, which are numerous in St. Clair County, surveyed, and models made to scale of many of them. These are now in the scientific collections of this and many other countries.

The Patrick collection of prehistoric crania, together with much other matter of interest from these mounds, is now in the collection of the Missouri Historical Society in St. Louis. For the past fifteen years the doctor has interested himself more in the line of his profession, which he has enriched by many papers upon subjects pertaining to dentistry, as well as by the invention of numerous instruments and appliances which are in use the wide world over.

Dr. Patrick has contributed much to the advancement of the dental profession in this country, and especially in the West and South, where few men were better known, and none more heartily welcomed.

The doctor was taken sick in January, 1894, but rallied from this, and on July 17 was apparently well, when he was taken with sciatic rheumatism, and other complications followed.

He was married March 15, 1895, to Miss Annie Rischer. Besides a wife, he leaves a brother, Dr. Hugh Patrick, in Lyons, France, and another, Andrew Patrick, at Liberty, Mo.

Dr. A. H. Fuller, in the *Western Dental Journal.*

